# The Diverse Major Histocompatibility Complex Haplotypes of a Common Commercial Chicken Line and Their Effect on Marek’s Disease Virus Pathogenesis and Tumorigenesis

**DOI:** 10.3389/fimmu.2022.908305

**Published:** 2022-05-27

**Authors:** Luca D. Bertzbach, Clive A. Tregaskes, Rebecca J. Martin, Undine-Sophie Deumer, Lan Huynh, Ahmed M. Kheimar, Andelé M. Conradie, Jakob Trimpert, Jim Kaufman, Benedikt B. Kaufer

**Affiliations:** ^1^ Institute of Virology, Freie Universität Berlin, Berlin, Germany; ^2^ Department of Pathology, University of Cambridge, Cambridge, United Kingdom; ^3^ Institute for Immunology and Infection Research, School of Biological Sciences, University of Edinburgh, Edinburgh, United Kingdom; ^4^ Department of Poultry Diseases, Faculty of Veterinary Medicine, Sohag University, Sohag, Egypt; ^5^ Veterinary Centre for Resistance Research (TZR), Freie Universität Berlin, Berlin, Germany

**Keywords:** major histocompatibility complex (MHC), B9, B15, B19, B21, resistance, susceptibility, VALO

## Abstract

The major histocompatibility complex (MHC) is crucial for appropriate immune responses against invading pathogens. Chickens possess a single predominantly-expressed class I molecule with strong associations between disease resistance and MHC haplotype. For Marek’s disease virus (MDV) infections of chickens, the MHC haplotype is one of the major determinants of genetic resistance and susceptibility. VALO specific pathogen free (SPF) chickens are widely used in biomedical research and vaccine production. While valuable findings originate from MDV infections of VALO SPF chickens, their MHC haplotypes and associated disease resistance remained elusive. In this study, we used several typing systems to show that VALO SPF chickens possess MHC haplotypes that include B9, B9:02, B15, B19 and B21 at various frequencies. Moreover, we associate the MHC haplotypes to MDV-induced disease and lymphoma formation and found that B15 homozygotes had the lowest tumor incidence while B21 homozygotes had the lowest number of organs with tumors. Finally, we found transmission at variable levels to all contact birds except B15/B21 heterozygotes. These data have immediate implications for the use of VALO SPF chickens and eggs in the life sciences and add another piece to the puzzle of the chicken MHC complex and its role in infections with this oncogenic herpesvirus.

## Introduction

Chickens vastly contributed to our understanding of the major concepts in immunology, genetics, virology and cancer (1–4). Longstanding research in avian immunology showed that the chicken BF-BL region, which is the major histocompatibility complex (MHC) and part of the larger B locus, exerts major effects on the immune responses of chickens towards various pathogens including viruses, bacteria and parasites (1, 4–6). The B locus is a cluster of genes located on chromosome 16 (5–7) and interestingly, chickens only express a single predominantly-expressed class I molecule from the BF2 gene (6, 8, 9). Chicken MHC haplotypes are strongly associated with resistance and susceptibility towards a large number of infectious diseases, including Marek’s disease virus (MDV) (5, 6, 10).

MDV is an oncogenic alphaherpesvirus that causes T cell lymphoma in chickens contributing to high economic losses in poultry farming (11, 12). Certain chicken MHC haplotypes have been associated with resistance and susceptibility to MDV. For example, chickens with the B21 haplotype are more resistant to MDV infections than B19 (13). It has been suggested that the MHC haplotypes can be ranked in terms of MDV susceptibility and resistance, although not all researchers agree on the degree and exact order (14, 15). Moreover, MHC haplotypes also influence the efficacy of vaccinations against MDV (16). Most of these data originate from experimental infections of specific pathogen free (SPF) chickens.

The German company VALO BioMedia is one of the largest producers of SPF eggs. VALO SPF eggs and chickens are widely used in vaccine production and avian infectious diseases research in general (17–27) including MDV (28–36) (references are not exhaustive and could have included many more recent publications). Despite their extensive use, the MHC haplotypes of VALO SPF chickens have not been reported previously.

In this study, we identified the MHC haplotypes of VALO SPF chickens used in previously published animal experiments (31–34, 37–39) and assessed the link between the MHC haplotypes and disease outcome. The MHC haplotypes were identified by a well-characterized microsatellite typing system (40–42) along with high through-put typing of class I and class II genes by amplification and next-generation sequencing (PCR-NGS), confirmed with haplotype-specific PCRs. These data allowed us to assess if the MHC haplotypes influence the onset of disease and tumorigenesis in VALO SPF chickens infected with the very virulent MDV strain RB-1B. Our data reveal that certain VALO SPF MHC haplotypes correlate with MDV resistance in both experimentally infected animals injected with the virus and animals infected *via* the natural route of infection by contact-exposure (31–34, 37, 39). Thus, this report sheds light on the influence of MHC haplotypes on MDV pathogenesis in VALO SPF chickens and provides valuable information on the MHC genetics for future biomedical research using this bird line.

## Materials and Methods

### Ethics Statement

All animal work was conducted in compliance with relevant national and international guidelines for care and humane use of animals. Experiments were approved by the Landesamt für Gesundheit und Soziales (LAGeSo) in Berlin, Germany (approval numbers G0218/12 and G0294/17).

### Cells and Viruses

Chicken embryo cells (CEC) were isolated and maintained as described previously (43). The very virulent MDV strain RB-1B (GenBank accession number MT797629) (39) was reconstituted by bacterial artificial chromosome (BAC) DNA transfection into CEC (44). Virus stocks were propagated in passaged CEC, frozen in liquid nitrogen and titrated on fresh CEC prior to the infection of the animals. Only low passage viruses were used (passage 4 to 7).

### Animal Experiments

One-day old VALO SPF chickens (n=88; from VALO BioMedia; Osterholz-Scharmbeck, Germany) were infected with RB-1B as previously described (31–34, 37–39) (and in as yet unpublished experiments). In addition, age-matched contact chickens (n=55) were co-housed to assess natural virus transmission. Water and food were provided *ad libitum*. Notably, all experiments were performed following standard protocols and in a climate-controlled BSL-2 environment. Blood samples were taken post infection from the brachial vein for subsequent DNA extractions. All chickens were assessed every day of the 13-week experiments to monitor MDV-specific clinical signs (to determine Marek’s disease incidences). Once clinical symptoms appeared or at termination of the experiments, chickens were euthanized and thoroughly examined for tumor lesions (to assess tumor incidences).

### DNA Extractions

DNA samples were isolated from 10µl whole blood using the E-Z96 blood DNA kit (Omega Bio-Tek; Norcross, GA, USA) according to the manufacturer’s instructions.

### MHC Typing

The use of the microsatellite LEI0258 to identify chicken MHC haplotypes is well-established, including for MDV studies (40–42). The correlation of the microsatellite typing to gene sequences became possible by use of a new high through-put PCR-NGS typing system (C. A. Tregaskes et al., manuscript in preparation). In brief, PCR from genomic DNA from 330 chickens using bar-coded primers amplified fragments from exon 2 to exon 3 (including the intron in between) of the MHC genes BLB1, BLB2, BF1 and BF2, which were ligated to bar-coded Illumina primers. A library of these double bar-coded amplicons was subjected to end-sequencing with a MiSeq instrument (Illumina; San Diego, CA, USA), and the resulting sequences were analyzed by a bespoke bioinformatics pipeline. The sequences identified by PCR-NGS typing for the five MHC haplotypes were identical [in exons 2 for BLB and exons 2 and 3 for BF as described in (45)] to the previously published sequences with the GenBank accession numbers (in parentheses) as follows: B9 (AB426145), B9:02 (AF539401, AF099115, AY489146, AF094778), B15 (AB426149, AM282695), B19 (AL023516, AM279338, AM282696), and B21 (AB426152, AM279337, AM282697). In order to confirm the assignments by PCR-NGS, the same samples of genomic DNA were amplified using haplotype-specific primers for BF2 gene alleles (L. Huynh et al., manuscript in preparation).

### Statistical Analyses

Statistical analyses were performed in Graph-Pad Prism v9 (GraphPad Software, San Diego, CA, USA). All statistical tests can be found in the respective figure legends. Data were considered significantly different if p ≤ 0.05.

## Results

The aim of this study was to assess the association of MHC haplotypes in VALO SPF chickens to the onset of Marek’s disease, overall tumor incidence and metastatic spread of MDV-induced tumors in experimentally infected and contact chickens of this chicken line.

We sequenced and analyzed DNA of 330 VALO SPF chickens primarily from previously published MDV infection experiments (31–34, 37–39) using an established microsatellite marker, a new PCR-NGS typing method and by haplotype-specific PCRs.

**Figure 1:**
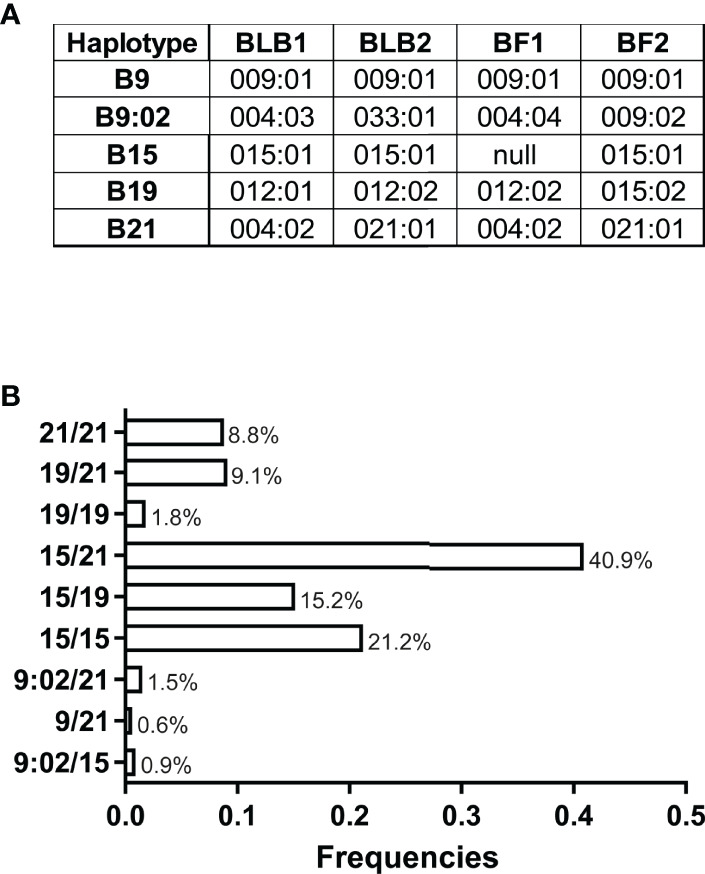
**(A)** Alleles of BLB1, BLB2, BF1 and BF2 genes found as haplotypes in VALO SPF chickens, correlated with standard names of MHC haplotypes, except for B9:02 named after the BF2 allele, which is a variant of the BF2 allele in the standard B9 haplotype. **(B)** Frequency distribution of MHC haplotypes in these batches of VALO SPF chickens (n = 330).

We found five MHC haplotypes in these VALO SPF chickens: B9, B9:02, B15, B19 and B21. The nomenclature of the gene alleles and MHC haplotypes used here is based on the latest attempts to develop a unified nomenclature (45), which has not been officially accepted (**Figure 1A**). However, three of the haplotypes found (B9, B15 and B21) have gene sequences identical to established standard MHC haplotypes (46). In addition, the B19 haplotype has the BF2 sequence originally described, which was re-named as B19var1 for some years, but more recently is clearly understood as the real B19 haplotype (45–47). The fifth haplotype (B9:02) has not been reported previously, but has been found widely in commercial egg-laying flocks (C. A. Tregaskes et al., manuscript in preparation), and was named with the accepted convention that the BF2 allele would define the B haplotype (46).

We show the haplotype frequencies for these VALO SPF chickens, finding that B15, B19 and B21 are the most frequent. While the heterozygous genotype B15/21 dominates in frequencies, we also identified considerable percentages of heterozygous B15/19 and B19/21 genotypes as well as homozygous B15 and B21. Very few chickens were homozygous for B19 and none for B9 and B9:02 in this cohort (**Figure 1B**).

**Figure 2:**
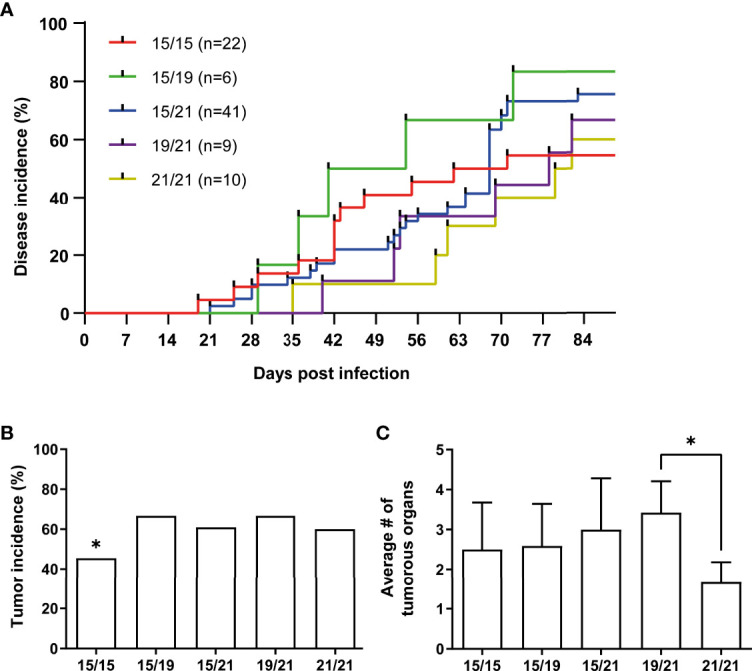
**(A)** Kaplan-Meier analysis of Marek’s disease incidence (scored as clinical signs of the disease) in wild type virus infected chickens with different MHC haplotypes (n.s., Mantel–Cox test; n = 88). **(B)** Tumor incidences in infected chickens with different MHC haplotypes as percentage of chickens with macroscopic tumors from all chickens that were experimentally infected (*p ≤ 0.5 indicates significant difference of B15/15 to all other haplotypes, Chi-square test; n = 88). **(C)** Mean number of affected organs harboring gross tumors per infected chicken (*p ≤ 0.5, Kruskal-Wallis test with a Dunn’s multiple comparison *post-hoc* test; n = 51).

**Figure 3:**
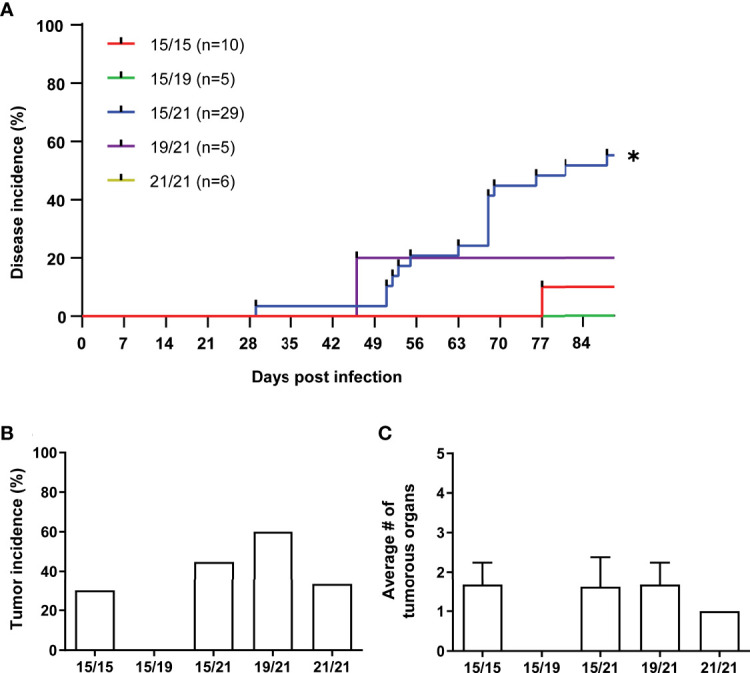
**(A)** Kaplan-Meier analysis of Marek’s disease incidence in contact chickens with different MHC haplotypes (*p ≤ 0.5, Mantel–Cox test; n=55). **(B)** Tumor incidences in contact chickens with different MHC haplotypes as percentage of chickens with macroscopic tumors (n.s., Chi-square test; n=55). **(C)** Mean number of affected organs harboring gross tumors per contact chicken (n.s., Kruskal-Wallis test with a Dunn’s multiple comparison *post-hoc* test; n=21).

To investigate associations of MHC haplotypes to MDV tumors, we then analyzed all wild type RB-1B-infected chickens (n=88) and their co-housed naïve contacts (n=55) of our cohort for Marek’s disease incidence, tumor incidence as well as the numbers of tumor-containing organs per animal (**Figures 2**, **3**). A Kaplan-Meier analysis of Marek’s disease incidence in infected chickens with different MHC haplotypes revealed no obvious differences in disease development, although homozygous B15 chickens had the lowest number of diseased chickens at the end of the studies. In addition, the median survival times of homozygous B21 chickens was the highest among all groups with 80.5 days (**Figure 2A**). Regarding the overall tumor incidences in infected chickens, we could show that homozygous B15 had the lowest tumor incidence among all the VALO SPF chickens, while the frequency of macroscopic tumors is roughly the same for the other haplotype combinations (**Figure 2B**). Finally, the assessment of overall affected tumorous organs to elucidate the impact of MHC haplotypes on tumor dissemination demonstrates some additional differences between the groups. In B21 homozygotes, the average number of tumorous organs per chicken was reduced compared to B15/15 and heterozygous genotypes B15/19, B15/21 and significantly compared to B19/21 (**Figure 2C**), which is in line with our data on disease incidence and a delayed disease onset (**Figure 2A**). These trends were also detected in contact chickens, where Marek’s disease signs were absent in B21/21 and B15/19 chickens and tumors in B21/21 chickens only appeared at final necropsy (**Figures 3A, B**). Interestingly, disease incidence in heterozygous B15/21 chickens was significantly increased in contact chickens (**Figure 3A**). The average number of tumorous organs per chicken was overall reduced in the contact chickens and we also found less tumors in the B21/21 group (**Figure 3C**).

## Discussion

In this report, we show that haplotype frequencies in VALO SPF chickens vary and include B9, B9:02, B15, B19 and B21 haplotypes, some of which are known to be quite commonly encountered haplotypes in layers (45, 48, 49). This haplotype diversity is likely due to the outbred nature of VALO SPF chickens, which have undergone constant genetic selection on performance traits since the 1960s (50). Our findings on two different disease phenotypes, Marek’s disease as well as tumor incidences of infected chickens, contradict previous reports that found high Marek’s disease susceptibility in B15 homozygotes (51, 52). However, a study in Hy-Line layers also described MDV resistant B15 chickens (42), suggesting that other factors must also contribute to the resistance against MDV. Our findings underline this resistant phenotype of layers in general and particularly of VALO SPF B15 and B21 homozygotes. It is important to note that increased susceptibility in heterozygous layers compared to B21 homozygotes has also been observed in previous experiments (48, 53, 54).

Our report is the first to characterize the VALO SPF MHC haplotype diversity and its impact on Marek’s disease and MDV-induced tumorigenesis in experimental infections. Our data indicate that several genes within the BF-BL region may affect different aspects of MDV pathogenesis in these chickens, as discussed previously (55, 56). In addition, while VALO SPF chicken MHC haplotypes influence disease parameters, it is likely that other unidentified non-MHC genes play important roles in host resistance to MDV infections that must have been selected for (42, 57–60). Notably, recent reports provided expression data of specific transcripts in VALO SPF chickens or improved avian RNAseq protocols (61, 62). However, to the best of our knowledge more detailed or even full genome and transcriptome data for VALO SPF chickens have not been published to date.

Overall, these data on VALO SPF chickens are valuable for their further use in avian immunology, avian infectious diseases research and vaccine production.

## Data Availability Statement

The raw data supporting the conclusions of this article will be made available by the authors, without undue reservation.

## Ethics Statement

The animal study was reviewed and approved by Landesamt für Gesundheit und Soziales (LAGeSo) Berlin.

## Author Contributions

LB, CT, JK and BK designed the study. LB, AK, AC and JT performed the animal experiments. CT, RM, U-SD and LH performed MHC typing. LB, CT, RM, JK and BK analyzed and interpreted the data. LB wrote the paper with input from JK, BK and all other authors. All authors contributed to the article and approved the submitted version.

## Funding

This work was funded by the DFG research unit grant “FOR 5130: ImmunoChick – Unravelling the avian immune response in the context of infection” awarded to JK and BK, a Wellcome Trust Investigator Award (110106/Z/15/Z) awarded to JK and the Volkswagen Foundation Lichtenberg grant A112662 awarded to BK.

## Conflict of Interest

The authors declare that the research was conducted in the absence of any commercial or financial relationships that could be construed as a potential conflict of interest.

## Publisher’s Note

All claims expressed in this article are solely those of the authors and do not necessarily represent those of their affiliated organizations, or those of the publisher, the editors and the reviewers. Any product that may be evaluated in this article, or claim that may be made by its manufacturer, is not guaranteed or endorsed by the publisher.
